# Abdominal pain and cirrhosis at diagnosis of hemochromatosis: Analysis of 219 referred probands with *HFE* p.C282Y homozygosity and a literature review

**DOI:** 10.1371/journal.pone.0261690

**Published:** 2021-12-21

**Authors:** James C. Barton, J. Clayborn Barton, Neha Patel, Gordon D. McLaren

**Affiliations:** 1 Department of Medicine, University of Alabama at Birmingham, Birmingham, AL, United States of America; 2 Southern Iron Disorders Center, Birmingham, AL, United States of America; 3 Division of Hematology/Oncology, Department of Medicine, University of California, Irvine, CA, United States of America; 4 Department of Veterans Affairs Long Beach Healthcare System, Long Beach, CA, United States of America; Cliniques Universitaires Saint-Luc, BELGIUM

## Abstract

**Background:**

In hemochromatosis, causes of abdominal pain and its associations with cirrhosis are poorly understood.

**Methods:**

We retrospectively compared characteristics of referred hemochromatosis probands with *HFE* p.C282Y homozygosity with/without biopsy-proven cirrhosis: sex, age, diabetes, heavy alcohol consumption, abdominal pain/tenderness, hepatomegaly, splenomegaly, non-alcoholic fatty liver disease, chronic viral hepatitis, ascites, transferrin saturation (TS), serum ferritin (SF), and iron removed by phlebotomy (QFe). We performed logistic regression on cirrhosis using characteristics identified in univariate comparisons. We performed computerized and manual searches to identify hemochromatosis case series and compiled prevalence data on cirrhosis and abdominal pain and causes of abdominal pain.

**Results:**

Of 219 probands, 57.1% were men. Mean age was 48±13 y. In 22 probands with cirrhosis, proportions of men, mean age, prevalences of heavy alcohol consumption, abdominal pain, abdominal tenderness, hepatomegaly, splenomegaly, and chronic viral hepatitis, and median TS, SF, and QFe were significantly greater than in probands without cirrhosis. Regression analysis revealed three associations with cirrhosis: abdominal pain (p = 0.0292; odds ratio 9.8 (95% CI: 1.2, 76.9)); chronic viral hepatitis (p = 0.0153; 11.5 (95% CI: 1.6, 83.3)); and QFe (p = 0.0009; 1.2 (95% CI: 1.1, 1.3)). Of eight probands with abdominal pain, five had cirrhosis and four had diabetes. One proband each with abdominal pain had heavy alcohol consumption, chronic viral hepatitis B, hepatic sarcoidosis, hepatocellular carcinoma, and chronic cholecystitis, cholelithiasis, and sigmoid diverticulitis. Abdominal pain was alleviated after phlebotomy alone in four probands. In 12 previous reports (1935–2011), there was a negative correlation of cirrhosis prevalence and publication year (p = 0.0033). In 11 previous reports (1935–1996), a positive association of abdominal pain prevalence and publication year was not significant (p = 0.0802).

**Conclusions:**

Abdominal pain, chronic viral hepatitis, and QFe are significantly associated with cirrhosis in referred hemochromatosis probands with *HFE* p.C282Y homozygosity. Iron-related and non-iron-related factors contribute to the occurrence of abdominal pain.

## Introduction

In 1871, hemochromatosis was defined as the syndrome of diabetes, hyperpigmentation, cirrhosis, and iron overload (’diabète bronzé et cirrhose pigmentaire’) [1]. In 1996, diagnostic criteria for hemochromatosis changed after the discovery that ~90% of non-Hispanic white adults with hemochromatosis are homozygous for the *HFE* (homeostatic iron regulator; chromosome 6p22.2) p.C282Y allele (exon 4, c.845G>A; rs1800562) [2,3] and that *HFE* p.C282Y homozygosity occurs in 0.3–0.6% of non-Hispanic whites [3]. Hepcidin expression by the liver is inappropriately low in many p.C282Y homozygotes and thus ferroportin internalization is also decreased, resulting in increased iron export by macrophages, elevated serum iron levels, and increased iron absorption despite increased iron stores [4,5]. Laboratory phenotypes typical of untreated p.C282Y homozygotes include elevated serum iron, transferrin saturation (TS), and serum ferritin (SF) levels [3,6].

Clinical penetrance of iron overload is mild in most *HFE* p.C282Y homozygotes [3,7,8]. Severe iron overload in some p.C282Y homozygotes is complicated by arthropathy, diabetes, other endocrinopathy, cirrhosis, primary liver cancer, and cardiomyopathy, of which cirrhosis and primary liver cancer are major complications [3]. Univariable analyses revealed that cirrhosis risk in persons with hemochromatosis and p.C282Y homozygosity is greater with age [9,10], male sex [10,11], diabetes [12], alcohol consumption [13], and severe iron overload [10,14]. These risk factors for cirrhosis were confirmed in a multivariable statistical model [15]. Blood donation [16,17] or chronic ingestion of oral iron supplements [18] may modify iron loading in p.C282Y homozygotes.

Abdominal pain was the most common symptom of patients with hemochromatosis at diagnosis reported in Sheldon’s 1935 monograph of 311 cases [19]. In 1955, Finch and Finch stated that "from 35 to 40 percent [of adults with hemochromatosis] develop significant abdominal pain sometime in the course of their disease…" [20]. During the interval 1935–1996, the prevalence of abdominal pain at diagnosis of 1832 patients with hemochromatosis was 35.6% (95% confidence interval (CI): 33.4, 37.8) in nine case series or compilations [3,19–26]. Finch and Finch also commented that, "In many of these patients it [abdominal pain] persists for months or years with remissions and exacerbations, while in others it may develop shortly before death" [20]. "In a few it has been associated with ascites, acute pancreatitis or perisplenitis" [20]. In another report, four patients with hemochromatosis and abdominal pain at diagnosis had peptic ulcer, three had variceal bleeding, three others had ascites, and one had nephrolithiasis [24]. Thus, there are multiple clinical correlates of abdominal pain in adults with hemochromatosis, although "The etiology of abdominal pain in hemochromatosis is poorly understood" [20].

To learn more about causes of abdominal pain at diagnosis and their associations with cirrhosis in hemochromatosis, we performed a retrospective evaluation of 219 consecutive, unselected referred hemochromatosis probands with *HFE* p.C282Y homozygosity diagnosed and treated at a single referral center. We evaluated associations of abdominal pain at diagnosis, cirrhosis, and other co-morbid factors in our case series and reviewed associations of abdominal pain and cirrhosis in hemochromatosis in previous reports. We discuss clinical associations of abdominal pain and cirrhosis in hemochromatosis.

## Methods

### Ethics statement

Western Institutional Review Board granted an exemption determination for performance of this study under 45 CFR 46.101(b)(4) (submission no. 2539985–44189619). Western Institutional Review Board waived the requirement to obtain informed consent because this study involved retrospective chart review and analyses of observations recorded in routine medical care. Data reported in patient-related observations were compiled from medical records as described in detail below and were compiled in a manner that maintained anonymity in datasets and results displayed in this study. This study did not involve the direct analysis of biological specimens, images, or questionnaires.

### Subjects included

We compiled observations on 219 consecutive self-identified non-Hispanic white adults aged ≥18 y who were referred to an Alabama tertiary center (1990–2018) for evaluation of hemochromatosis and who were *HFE* p.C282Y homozygotes and the first in their respective families to be diagnosed to have hemochromatosis (probands). No proband was previously diagnosed to have or treated for hemochromatosis. A history of current and past medical events was recorded from probands and records provided by their referring physicians. All probands underwent pertinent physical examination and laboratory testing, including evaluation of abdominal manifestations and liver conditions, before treatment was initiated. Liver biopsy specimens were obtained as recommended in diagnosis and management guidelines for hemochromatosis of the American Association for the Study of Liver Diseases [27]. All probands with abdominal pain at diagnosis underwent CT scanning of the abdomen/pelvis. Other radiographic imaging procedures, endoscopy, and blood tests were also performed to identify cause(s) of abdominal pain, as appropriate.

### Subjects excluded

We excluded *HFE* p.C282Y homozygotes who: were family members of the present probands; presented with iron deficiency (both TS <10% and SF <15 μg/L); were treated with phlebotomy or iron chelation before referral; or who had incomplete evaluations. We also excluded patients with hemochromatosis who were previously diagnosed as participants in the Hemochromatosis and Iron Overload Screening (HEIRS) Study [6,28].

### Definition of pain

We defined pain according to the revised International Association for the Study of Pain: "an unpleasant sensory and emotional experience associated with, or resembling that associated with, actual or potential tissue damage" [29].

### Diabetes

Diabetes was diagnosed by referring physicians and classified according to criteria of the American Diabetes Association [30].

### Abdominal manifestations

Abdominal pain at diagnosis was reported as chronic or intermittent pain or discomfort between the diaphragm and pelvis unassociated with trauma or obvious non-trauma causes (e.g., gastroesophageal reflux, constipation). Abdominal tenderness was defined as proband comment of pain during abdominal examination. Hepatomegaly was defined as liver >15 cm in vertical span in the right mid-clavicular line determined by percussion and palpation or >16 cm by ultrasonography or CT scanning [31]. Splenomegaly was defined as dullness in Traube’s space, palpable spleen tip on deep inspiration, or spleen >13 cm in vertical span by ultrasonography or CT scanning [32]. Ascites suspected by history or on physical examination was confirmed by abdominal ultrasonography or CT scanning. All physical examinations were performed by a single author (JaCB).

### Liver conditions

Heavy ethanol consumption was defined as the self-reported estimated consumption of ≥60 g ethanol/d for ≥5 y. Non-alcoholic fatty liver disease (NAFLD) was defined as steatosis or steatohepatitis detected on liver specimens obtained by biopsy or as diffuse or focal increased hepatic echogenicity detected by ultrasonography, in the absence of self-reports of heavy ethanol consumption. Chronic viral hepatitis B or C was defined as positivity for hepatitis B surface antigen (HB_s_Ag) or hepatitis C antibody, respectively, in association with other clinical or histologic liver abnormalities consistent with chronic viral hepatitis. Liver biopsy was performed in all probands with SF >1000 μg/L and was recommended to other probands whose evaluations suggested that they had a liver condition other than iron overload. Pathologists interpreted liver specimens and defined cirrhosis as the histological occurrence of regenerating nodules of hepatocytes surrounded by bands of fibrous connective tissue [33]. Iron staining in liver specimens was graded 0–4+ as previously described [34].

### Iron removed by phlebotomy (QFe)

Iron depletion therapy, defined as the periodic removal of blood to eliminate storage iron, was performed in men with SF >300 μg/L and in women with SF >200 μg/L. Therapy was complete when SF was ≤20 μg/L [35]. QFe was estimated to be 200 mg Fe per unit of blood (450–500 mL) [35].

### Laboratory methods

TS, SF, HB_s_Ag, and hepatitis C antibody were measured using standard clinical laboratory methods (Laboratory Corporation of America, Burlington, NC, USA). *HFE* allele analyses were performed as previously described [36].

### Literature review

The authors performed computerized searches of the National Library of Medicine and the internet and manual searches of printed texts and articles using the terms hemochromatosis, abdominal pain, and cirrhosis, and recorded prevalence data of abdominal pain and cirrhosis in case series or compilations of hemochromatosis patients diagnosed in referral venues. The date range of the literature search was 1871 [1] to 2020. We reviewed published reports to identify well-documented causes of abdominal pain in patients with hemochromatosis. We excluded reports of individual cases or families, clinical series that did not report percentages of all cases with abdominal pain, and case series of pathological evaluations.

### Statistical analysis

The sample size of 219 probands represented the entirety of referred Alabama probands with hemochromatosis and *HFE* p.C282Y homozygosity whose medical records met all other stipulations for evaluation as described in *Subjects included*. Initial data exploration revealed that the proportion of probands who reported having abdominal pain was 3.7% (8/219) and the proportion of probands with cirrhosis who also reported abdominal pain was 22.7% (5/22), consistent with previous reports [20,37]. Accordingly, we performed univariate comparisons of probands with and without biopsy-proven cirrhosis regarding the following characteristics: sex, age at diagnosis, diabetes, heavy alcohol consumption, abdominal pain, abdominal tenderness, hepatomegaly, splenomegaly, NAFLD, chronic viral hepatitis, ascites, TS, SF, and QFe.

Descriptive data are displayed as enumerations, means ± 1 standard deviation (SD), medians (range), and percentages. Age data are expressed as the nearest whole year. Hemoglobin, TS, and SF data are expressed as the nearest integer. We evaluated continuous data for normality using d’Agostino’s and Shapiro-Wilk tests. Mean values of data from normal distributions were compared using Student’s t test (two-tailed). Differences in median values of data from non-normal distributions were compared using the Mann-Whitney U test. We compared differences in proportions of dichotomous variables using Fisher’s exact test (two-tailed) or Pearson’s Chi-square test with Yates’ correction (two-tailed), as appropriate. We compared some continuous data using Pearson’s correlation coefficient. CIs of proportions (95%) are displayed with continuity corrections. For univariate comparisons, we did not use a Bonferroni correction because most data were not positively correlated and we did not wish to overlook positive results [38].

We performed logistic regression on cirrhosis using characteristics that differed between probands with and without cirrhosis in univariate comparisons (p ≤0.1000) [39], except that we excluded SF because SF is significantly correlated with liver iron content [40] and QFe [41]. We computed odds ratios (OR) and 95% CI for significant independent variables. Values of p <0.05 were defined as significant. Analyses were performed with Excel 2000^®^ (Microsoft Corp., Redmond, WA, USA), GB-Stat^®^ (v. 10.0, 2003; Dynamic Microsystems, Inc., Silver Spring, MD, USA), and GraphPad Prism 8^®^ (2018; GraphPad Software, San Diego, CA, USA).

## Results

### General characteristics

There were 219 probands (125 men (57.1%) and 94 women (42.9%)). Mean age at diagnosis was 48 ± 13 y. Diabetes and heavy alcohol consumption, independent risk factors for cirrhosis in *HFE* p.C282Y homozygotes [15], occurred in 15.1% and 6.8% of probands, respectively. Eight probands (3.7%; 5 men, 3 women) had abdominal pain. Prevalence of other manifestations included the following: abdominal tenderness 3.2%, hepatomegaly 15.5%, splenomegaly 2.3%, NAFLD 22.4%, chronic viral hepatitis 3.6%, and ascites 0.9%. Seventy-eight probands (78/219; 35.6%) underwent liver biopsy, of whom 22 had cirrhosis (22/219; 10.0%). Mean hemoglobin was 146 ± 15 g/L. Median TS was 81% (range 19–100). Median SF was 712 μg/L (range 15–6103). Median QFe was 2.8 g (range 0, 31.2).

### Univariate comparisons

The proportion of men was significantly greater among probands with than probands without cirrhosis (Table 1). Mean age of probands with cirrhosis was significantly greater than that of probands without cirrhosis (Table 1). The respective prevalences of heavy alcohol consumption, abdominal pain, abdominal tenderness, hepatomegaly, splenomegaly, and chronic viral hepatitis were significantly greater in probands with than without cirrhosis. Median TS, SF, and QFe values were significantly greater in probands with than without cirrhosis (Table 1).

**Table 1:** Characteristics of 219 hemochromatosis probands with *HFE* p.C282Y homozygosity^a^.

Characteristic	Cirrhosis (n = 22)	No cirrhosis (n = 197)	Value of p
Male, % (n)	86.4 (19)	53.8 (106)	0.0030
Mean age at diagnosis, y, ± 1 SD	52 ± 9	48 ± 14	0.0451
Diabetes, % (n)^b^	22.7 (5)	14.2 (28)	0.3417
Heavy alcohol consumption, % (n)	22.7 (5)	5.1 (10)	0.0099
Abdominal pain, % (n)	22.7 (5)	1.5 (3)	0.0003
Abdominal tenderness, % (n)	13.6 (3)	2.0 (4)	0.0238
Hepatomegaly, % (n)	45.5 (10)	12.2 (24)	0.0004
Splenomegaly, % (n)	13.6 (3)	1.0 (2)	0.0078
NAFLD, % (n)	27.3 (6)	21.8 (43)	0.5914
Chronic viral hepatitis, % (n)^c^	18.2 (4)	1.5 (3)	0.0022
Ascites, % (n)	4.8 (1)	0.5 (1)	0.1912
Mean hemoglobin, g/L, ± 1 SD	140 ± 18	147 ± 15	0.1559
Median TS, % (n) (range)	89 (34, 100)	70 (19, 100)	0.0235
Median SF, μg/L (range)	2000 (387, 5613)	650 (15, 6103)	<0.0001
Median QFe, g (range)	6.3 (1.0, 31.2)	2.6 (0, 30.0)	<0.0001

^a^Abbreviations: NAFLD, non-alcoholic fatty liver disease; QFe, quantity of iron removed by phlebotomy to achieve iron depletion; SD, standard deviation; SF, serum ferritin; TS, transferrin saturation.

^b^Thirty-one probands had type 2 diabetes. Two other probands had type 1 diabetes.

^c^Five probands had chronic viral hepatitis C. Two other probands had chronic viral hepatitis B. No proband was treated with anti-viral therapy for chronic viral hepatitis before diagnosis of hemochromatosis.

Among 22 probands with cirrhosis, the proportion of men was significantly lower in those with than without abdominal pain, although the prevalence of co-morbid conditions in probands with cirrhosis with and without abdominal pain did not differ significantly (Table 2).

**Table 2:** Hemochromatosis probands with *HFE* p.C282Y homozygosity and cirrhosis^a^.

Characteristics	Abdominal pain (n = 5)	No abdominal pain (n = 17)	Value of p
Male, % (n)	60.0 (3)	94.1 (16)	0.0239
Mean age at diagnosis, y, ± 1 SD	50 ± 9	59 ± 7	0.0835
Diabetes, % (n)^b^	40.0 (2)	17.6 (3)	0.5431
Heavy alcohol consumption, % (n)	20.0 (1)	23.5 (4)	1.0000
Hepatomegaly, % (n)	40.0 (2)	47.1 (8)	1.0000
Splenomegaly, % (n)	20.0 (1)	11.8 (2)	1.0000
NAFLD, % (n)	20.0 (1)	29.4 (5)	1.0000
Chronic viral hepatitis, % (n)	0 (0)	23.5 (4)	0.5304
Ascites, % (n)	0 (0)	5.9 (1)	1.0000
Hepatic sarcoidosis, % (n)	20.0 (1)	0 (0)	0.2273

^a^ Abbreviations: SD, standard deviation; NAFLD, non-alcoholic fatty liver disease.

### Logistic regression on cirrhosis

We performed regression on cirrhosis using independent variables that were significantly associated with cirrhosis in univariate comparisons (male sex, age at diagnosis, heavy alcohol consumption, abdominal pain, abdominal tenderness, hepatomegaly, splenomegaly, chronic viral hepatitis, TS, and QFe) (Table 1). This regression revealed three positive associations: abdominal pain (p = 0.0292; OR 9.8 (95% CI: 1.2, 76.9)); chronic viral hepatitis (p = 0.0153; OR 11.5 (95% CI: 1.6, 83.3)); and QFe (p = 0.0009; OR 1.2 (95% CI: 1.1, 1.3)). ANOVA p of this regression was <0.0001. This model accounted for 36.8% of cirrhosis deviance.

### Eight probands with abdominal pain

Median TS, SF, and QFe of probands with and without abdominal pain did not differ significantly (Table 3).

**Table 3:** Abdominal pain in hemochromatosis probands with *HFE* p.C282Y homozygosity.

Iron-related measures	Abdominal pain (n = 8)	No abdominal pain (n = 219)	Value of p
Median TS, % (n) (range)	89 (39, 100)	81 (19, 100)	0.3735
Median SF, μg/L (range)	2036 (276, 5001)	709 (15, 6103)	0.0711
Median QFe, g (range)	5.8 (1.2, 16.0))	2.8 (0, 31.2)	0.0837

^a^Abbreviations: QFe, quantity of iron removed by phlebotomy to achieve iron depletion; SF, serum ferritin; TS, transferrin saturation.

Seven of eight probands with abdominal pain underwent liver biopsy. Each of the eight probands underwent CT scanning of the abdomen/pelvis and other evaluation to identify the cause(s) of abdominal pain and non-iron liver conditions (Table 4). Five of eight probands with abdominal pain also had cirrhosis (Table 4). Four of these eight probands also had diabetes. One proband each had chronic viral hepatitis B, heavy alcohol consumption, hepatic sarcoidosis, and hepatocellular carcinoma. Another proband with cirrhosis also had chronic cholecystitis, cholelithiasis, and sigmoid diverticulitis. Four probands reported that phlebotomy therapy alone alleviated abdominal pain. Three other probands achieved relief of abdominal pain with phlebotomy in combination with surgical or medical treatment. In a 51 year-old woman, no cause of abdominal pain was discovered nor did she achieve relief of abdominal pain after phlebotomy (Table 4).

**Table 4:** Hemochromatosis probands with *HFE* p.C282Y homozygosity and abdominal pain^a^.

Age/sex	Abdominal pain description	Liver and other conditions	Relief of pain	QFe, g
37M	general abdominal discomfort^b^	diabetes; 4+ liver iron; hepatic iron 26,115 μg/g dry weight; no cirrhosis	phlebotomy	6.8
47M	general abdominal discomfort^c^	hepatomegaly; 4+ liver iron; no cirrhosis; chronic viral hepatitis B	phlebotomy	5.6
51F	general abdominal discomfort^d^	diabetes; hepatomegaly; no other liver condition identified; no liver biopsy	no cause discovered; no relief with phlebotomy	1.2
52M	RUQ pain^e^	4+ liver iron; cirrhosis	cholecystectomy; sigmoid colon resection; phlebotomy	6.0
53M	RUQ pain, tenderness^f^	diabetes; heavy alcohol consumption; 4+ liver iron; cirrhosis	phlebotomy	3.0
58F	RUQ pain^g^	splenomegaly; 3+ liver iron; cirrhosis; hepatic sarcoidosis; NAFLD	treatment of sarcoidosis; phlebotomy	1.4
62F	RUQ pain, tenderness^h^	diabetes; 4+ liver iron; cirrhosis	phlebotomy	12.0
67M	RUQ pain^i^	hepatomegaly; 4+ liver iron; cirrhosis; hepatocellular carcinoma	resection of hepatocellular carcinoma; phlebotomy	16.0

^a^Abbreviations: EGD, esophagogastroduodenoscopy; EKG, electrocardiogram; NAFLD, non-alcoholic fatty liver disease; RUQ, right upper quadrant; US, abdominal ultrasonography; QFe, quantity of iron removed by phlebotomy to achieve iron depletion.

^b^Evaluation included CT scan of abdomen/pelvis, EGD, and gastric and small bowel biopsy.

^c^Evaluation included CT scan of abdomen/pelvis, EKG, and echocardiogram.

^d^Evaluation included CT scan of abdomen/pelvis, US, EGD, and cardiac monitoring.

^e^Evaluation included CT scan of abdomen/pelvis, US, and laparoscopic cholecystectomy (chronic cholecystitis, cholelithiasis), and sigmoid colectomy (sigmoid diverticulitis).

^f^Evaluation included CT scan of abdomen/pelvis, liver/spleen scan, US, and EGD.

^g^Evaluation included CT scan of abdomen/pelvis, EGD, and colonoscopy.

^h^Evaluation included CT scan of abdomen/pelvis, EGD, hepatobiliary scan, cholangiogram, and visceral arteriography.

^i^Evaluation included CT scan of abdomen/pelvis, US, alpha-fetoprotein level, electrocardiogram, and resection of hepatocellular carcinoma.

### Literature review

**Fig 1:**
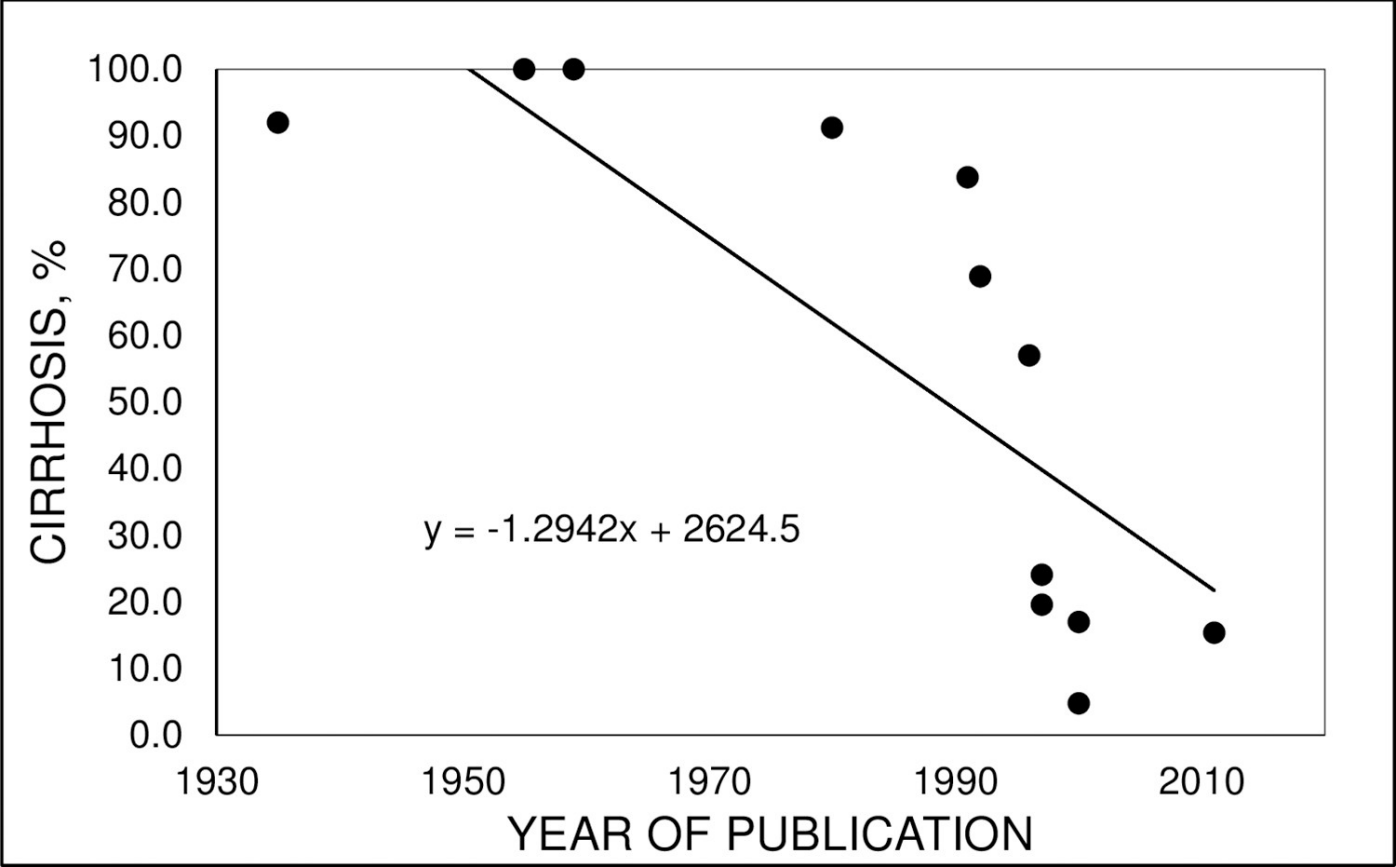
Hemochromatosis and cirrhosis. Percentages of adults with hemochromatosis diagnosed in non-screening venues who had cirrhosis in 12 reports [19,22,24–26,42–48]. Greater proportions of men than women had cirrhosis in all reports. Pearson correlation coefficient -0.7969; adjusted r^2^ = 0.5945 (p = 0.0033).

The prevalence of cirrhosis in patients with hemochromatosis diagnosed in non-population screening venues in 12 reports published in the interval 1935–2011 [19,22,24–26,42–48] is displayed in Fig 1. The median percentage of adults with hemochromatosis and cirrhosis was significantly greater during the publication interval 1935–1996 than the publication interval 1997–2011 (91.6% (range 57.0, 100.0) vs. 17.0% (range 4.8, 24.1), respectively; p = 0.0062).

**Fig 2:**
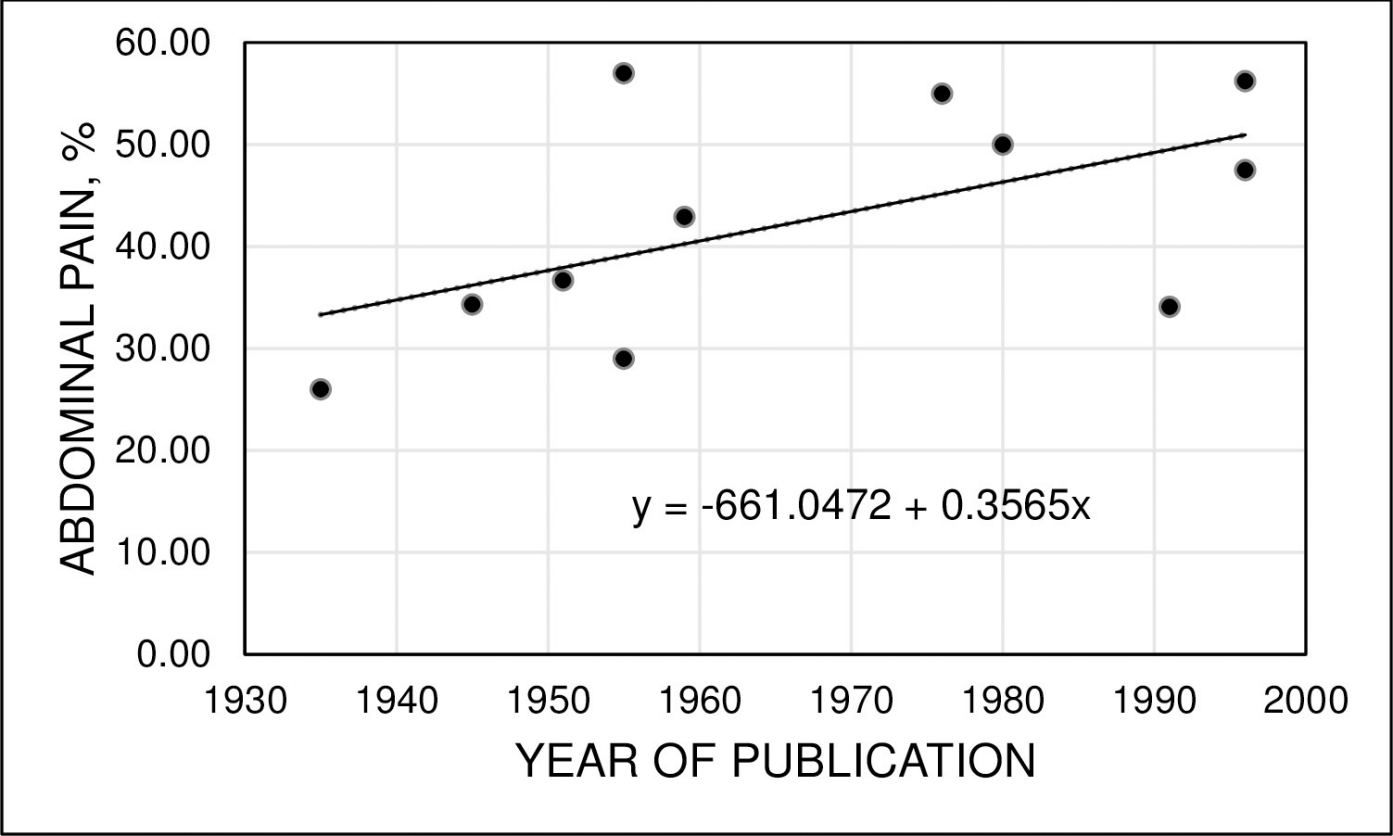
Hemochromatosis and abdominal pain. Percentages of adults with hemochromatosis diagnosed in referral venues who reported abdominal pain at diagnosis in 11 reports published during the interval 1935–1996 [3,19–26,42,49]. Pearson correlation coefficient 0.5491; adjusted r^2^ = 0.2239 (p = 0.0802).

In 11 reports published during the interval 1935–1996, there was a positive association of percentages of patients with hemochromatosis diagnosed in non-population screening venues who reported abdominal pain at diagnosis [3,19–26,42,49], although this association was not statistically significant (Fig 2).

### Conditions associated with abdominal pain

The present study and twelve previous reports, including some cited in Fig 2, revealed multiple well-documented causes of abdominal pain in adults with hemochromatosis (Table 5).

**Table 5:** Conditions associated with abdominal pain in adults with hemochromatosis.

Condition	Comments	References
*Likely associated with iron overload*		
Cirrhosis	in patients with abdominal pain, odds ratio for cirrhosis 9.8 (95% CI: 1.2, 76.9)^a^	present study
Iron overload	pain mechanism unknown; iron overload and cirrhosis often present; pain sometimes alleviated with phlebotomy alone	present study
Primary liver cancer	typically older men with iron overload and cirrhosis^b^; pain usually in right upper quadrant; pain relief with indicated surgical or medical treatment + phlebotomy	present study; [20,24,42,49,50]
Portal vein thrombosis	men with iron overload, epigastric or right upper quadrant pain^c^	[51,52]
Spontaneous *E*. *coli* peritonitis	iron overload and cirrhosis typical; rapidly progressive, severe abdominal pain, fever, and death ≤5 d after presentation^d^	[53–58]
Ascites	usually due to cirrhosis; pain more likely with large volumes	[20,24]
*Not likely associated with iron overload*		
Acute or chronic cholecystitis, cholelithiasis	right upper quadrant pain relief with indicated surgical or medical treatment + phlebotomy	present study; [24,42,59]
Hepatic sarcoidosis	cholestasis, extra-hepatic lesions common; right upper quadrant pain relief expected with corticosteroid treatment + phlebotomy	[60–62]
Sigmoid diverticulitis	pain relief with indicated surgical or medical treatment + phlebotomy	present study
Peptic ulcer	pain relief expected with indicated surgical or medical treatment + phlebotomy	[24,42]
Other conditions	abdominal pain has been attributed to nephrolithiasis, perisplenitis, acute pancreatitis, and diabetic neuropathy^e^	[20,24]
No demonstrable abnormality	risk of unexplained abdominal pain is higher in women than men unselected for hemochromatosis	present study; [11]

^a^Causes of cirrhosis in the present patients include iron overload, excessive alcohol consumption, chronic viral hepatitis B, and hepatic sarcoidosis.

^b^One patient with hemochromatosis, cirrhosis, and primary liver cancer presented with bleeding esophageal varices and portal vein thrombosis [63]. Another patient with hemochromatosis, cirrhosis, and primary liver cancer presented with spontaneous *E*. *coli* peritonitis [55].

^c^One patient had *HFE* p.C282Y/p.H63D and hepatic steatosis [51].

^d^Most patients with hemochromatosis and spontaneous *E*. *coli* peritonitis had heavy iron overload and cirrhosis [53–58]. One patient with hemochromatosis died of tuberculous peritonitis [42]. Another patient with hemochromatosis was diagnosed to have peritonitis not otherwise specified [49]. Other adults with hemochromatosis suffered from severe iron overload, and severe, progressive abdominal pain, followed by brief illnesses suggestive of spontaneous bacterial peritonitis leading to death, without positive bacterial cultures [37,64].

^e^We found no reports of persons with hemochromatosis who also had diabetes and gastroparesis.

## Discussion

A novel observation of this study is that, in non-screening hemochromatosis probands with *HFE* p.C282Y homozygosity, there is a significant positive statistical association between abdominal pain and cirrhosis, after adjustment for other variables. These findings substantiate and extend previous informal associations of abdominal pain with cirrhosis and its complications in adults with hemochromatosis, as reported in other case series or compilations [3,19–23,25,26,65]. Evaluations to determine causes of abdominal pain at presentation in the present probands varied. Thus, it is plausible that some probands with or without cirrhosis had abdominal pain due to or exacerbated by conditions that would not have been diagnosed without more exhaustive evaluations. Results of the present study also confirm previous reports of significant statistical associations of cirrhosis with chronic viral hepatitis [66] and QFe [10,14,15] in adults with hemochromatosis and p.C282Y homozygosity, after adjustment for other variables.

Sympathetic innervation of Glisson’s capsule and hepatic veins accounts for pain that arises in the liver [67]. Regardless of its cause, liver pain is often described as ill-defined, dull, or aching and its severity is typically reported as mild or moderate [67]. Abdominal pain was localized to the right upper quadrant in five of eight probands in the present study. Three other probands reported having pain that they described as general abdominal discomfort. In another study of patients with hemochromatosis diagnosed in referral venues, it was reported that "Most frequently it [abdominal pain] is dull and boring, crampy, dragging or aching, and usually located in the epigastrium or right upper quadrant" [20]. In 17 other patients with hemochromatosis, the most common qualities and locations of abdominal pain were "a dull aching sensation in the right upper quadrant or epigastrium. Cramping epigastric pains were also noted, and two patients had pain referred to the costovertebral angles" [24]. Taken together, these observations indicate that abdominal pain in patients with hemochromatosis at diagnosis often occurs in the right upper quadrant or epigastrium, although the location, quality, and severity of abdominal pain vary among patients in this and previous reports.

Factors that could account for liver pain in the present patients with or without cirrhosis include the individual or combined hepatic inflammatory effects of excessive iron [68], chronic hepatitis B virus infection [69], alcohol-induced liver disease [70], sarcoidosis [62,71], or NAFLD [62,72], and distention or distortion of the liver caused by hepatomegaly, cirrhosis, portal hypertension [67], or hepatocellular carcinoma [72]. Elevated blood biomarkers of inflammation are also common in patients with cirrhosis [70].

None of the present 23 probands with hemochromatosis and diabetes was diagnosed to have gastroparesis. We found no reports of persons with hemochromatosis who also had diabetes and gastroparesis. In 134 patients with gastroparesis attributed to diabetes, 89% had abdominal pain, especially epigastric pain, although none was described to have hemochromatosis [73].

Abdominal pain was alleviated by phlebotomy therapy alone in three male probands and one female proband with iron overload in this study. Two of these men and the woman had cirrhosis. Three other probands, each of whom had cirrhosis, achieved relief of abdominal pain with surgical or medical treatment in combination with phlebotomy, although the relative contribution of phlebotomy to relief of abdominal pain in these probands, if any, is indeterminable. One woman without cirrhosis did not achieve abdominal comfort after phlebotomy therapy and no other cause of her abdominal pain was discovered. Altogether, factors associated with abdominal pain included iron overload in seven of eight probands and cirrhosis in six of eight probands. Although achieving iron depletion in *HFE* p.C282Y homozygotes with cirrhosis may alleviate abdominal pain, reversal of cirrhosis after therapeutic phlebotomy in persons with hemochromatosis is uncommon [74].

It is plausible but unproven that alleviation of abdominal pain was a placebo effect of phlebotomy in some of the present patients. Nonetheless, it is widely acknowledged that a study design in which patients with hemochromatosis and iron overload (with or without abdominal pain) are randomized to receive or not receive phlebotomy treatment (or other means to reduce iron overload) is unethical. Likewise, it is also unethical to study the outcomes of phlebotomy therapy to induce iron depletion in hemochromatosis patients (with or without abdominal pain) using control subjects without hemochromatosis (with or without abdominal pain) who undergo phlebotomy to the same extent as hemochromatosis patients. In a multicenter, participant-blinded study of hemochromatosis patients with *HFE* p.C282Y homozygosity and SF 300–1000 μg/L, participants were randomly assigned to undergo either iron reduction with erythrocytapheresis (treatment group) or sham treatment with plasmapheresis (control group) [75]. In the treatment group, the target of erythrocytapheresis was SF <300 μg/L [75]. Improvements in several subjective and objective measures occurred in treatment participants that were significantly greater than those in control participants, although no study participant was reported to have either abdominal pain or cirrhosis [75]. Performing a similar study of hemochromatosis patients with SF >1000 μg/L would not be ethical because there would be a risk of organ damage due to iron overload that remained untreated in control participants.

One male proband without cirrhosis in the present study had pain that he described as general abdominal discomfort and he also had hepatomegaly, chronic viral hepatitis B, and high QFe. In non-Asian patients in the US with chronic viral hepatitis B unselected for hemochromatosis, liver pain and abdominal tenderness occurred in ~70% and ~78%, respectively [69].

In the present study, hepatomegaly occurred in 34 of 219 probands (16%) and in three of eight probands with abdominal pain (38%). In six hemochromatosis case series or compilations published before the discovery of the *HFE* gene in 1996, the prevalence of hepatomegaly was 41–92% [19,20,25,26,43,76]. In the present study, hepatomegaly was not significantly associated with cirrhosis, after adjustment for other variables.

Primary liver cancer, typically hepatocellular carcinoma, is a complication of hemochromatosis, severe iron overload, and cirrhosis that occurs predominantly in older men [50,77]. In the present study, a 67 year-old man with right upper quadrant abdominal pain also had iron overload, cirrhosis, and hepatocellular carcinoma. In a 1955 compilation of 787 patients with hemochromatosis, it was reported that, "In 13 per cent of the cases studied it [abdominal pain] was accounted for on the basis of liver neoplasm” [20]. In a 1980 study, four of 17 patients with hemochromatosis and abdominal pain had hepatoma [24]. In a 1993 study, abdominal pain occurred in 15% of adults with hemochromatosis and primary liver cancer [50].

No cause for abdominal pain (described as general abdominal discomfort) or hepatomegaly was discovered in one female proband with hepatomegaly and low QFe in this study nor did she achieve relief of abdominal pain after phlebotomy. Abdominal pain of unknown etiology is more prevalent in women than men and laparotomy is unlikely to reveal a cause of abdominal pain in women who have had this symptom for more than three months [78].

Splenomegaly occurred in five of 219 probands (2%) and in one of eight probands with abdominal pain (13%). The prevalence of splenomegaly was significantly greater in probands with than without cirrhosis (14% vs. 1%, p = 0.0078), although splenomegaly was not associated with cirrhosis in a logistic regression, after adjustment for other variables. In four hemochromatosis case series or compilations published before the discovery of *HFE*, the prevalence of splenomegaly was 10–55% [19,20,26,43]. Variability in the prevalence of splenomegaly at diagnosis of hemochromatosis in adults in this and other reports may be due in part to the criteria used to define splenomegaly and to the severity and duration of iron overload, cirrhosis, and portal hypertension. In 910 patients with cirrhosis not otherwise specified, splenomegaly based on estimates of spleen size at diagnostic laparoscopy was present in 51% [79].

In a 2002 report of hemochromatosis screening performed in California health maintenance clinics, the proportions of 123 *HFE* p.C282Y homozygotes and 21,994 *HFE* wild-type control participants who reported abdominal pain on a questionnaire did not differ significantly (8.1% vs. 9.8%, respectively; p = 0.6393) [80]. In standardized post-screening clinical evaluations of participants in the North American primary-care based HEIRS Study that included questionnaires and focused physical examinations, the proportion of 195 previously undiagnosed p.C282Y homozygotes who reported "unexplained abdominal pain or discomfort in the last 12 months" was significantly greater than that of 364 *HFE* wild-type control participants (20.5% vs. 11.3%, respectively; p = 0.0037) [81]. Neither of these two screening studies reported causes of abdominal pain in study participants. Regardless, the proportions of p.C282Y homozygotes diagnosed by screening who reported abdominal pain are much lower than the aggregate 36% of 1832 referred patients with hemochromatosis who reported abdominal pain at diagnosis [3,19–26]. The aggregate prevalence of cirrhosis among p.C282Y homozygotes diagnosed in three population screening studies (2001–2006) [82–84] was also low (3.3% (95% CI: 2.3, 5.8)).

The present literature review demonstrates that the median percentage of adults with hemochromatosis who were diagnosed to have cirrhosis was significantly greater in articles published in the interval 1935–1996 than the interval 1997–2011. Each article describes a case series compiled over many years, like the present work. Thus, exact years of diagnosis of hemochromatosis are unreported or cannot be deduced for most individual patients. Nonetheless, results of our analysis are consistent with a shift of hemochromatosis diagnosis criteria from the triad “diabetes, bronzing, and cirrhosis” [1] to elevated TS, with or without hyperferritinemia, and a hemochromatosis-associated *HFE* genotype, especially p.C282Y homozygosity, after discovery of the *HFE* gene in 1996 [3]. Although we observed a positive relationship of year of publication with percentage of hemochromatosis patients with abdominal pain at diagnosis in case series, this relationship was not statistically significant. Interpretation of the latter relationship is limited because many hemochromatosis case series do not report observations on abdominal pain.

Strengths of this study include a large cohort of non-screening, unrelated adults with hemochromatosis and *HFE* p.C282Y homozygosity and a complete data set that includes histologically proven cirrhosis and QFe. A limitation of this study is that interpretation of abdominal pain by patients and physicians is subjective. Logistic regression on cirrhosis indicates that ~63% of the independent determinant(s) of cirrhosis in the present hemochromatosis probands were not identified or analyzed. Like the present probands, abdominal pain is common in adults unselected for *HFE* hemochromatosis, including those with [85–87] and without [88,89] cirrhosis. Although we did not compare characteristics of the present probands with those of other cohorts of adults with abdominal pain, the present study substantiates that the prevalence of abdominal pain at diagnosis in referred probands with *HFE* p.C282Y homozygosity is significantly greater among those with than among those without cirrhosis, after adjustment for other variables. Studying abdominal pain that occurred after diagnosis of hemochromatosis in the present probands was beyond the scope of the present study.

## Conclusions

We conclude that abdominal pain, chronic viral hepatitis, and QFe are significantly associated with cirrhosis at diagnosis in non-screening hemochromatosis probands with *HFE* p.C282Y homozygosity. Abdominal pain was uncommon in hemochromatosis probands without cirrhosis or diabetes. Iron-related and non-iron-related factors contribute to the occurrence of abdominal pain.

## Supporting information

S1 File (XLSX)
